# Low-hysteresis, pressure-insensitive, and transparent capacitive strain sensor for human activity monitoring

**DOI:** 10.1038/s41378-022-00450-7

**Published:** 2022-10-12

**Authors:** Xiaoyi Wang, Yang Deng, Peng Jiang, Xingru Chen, Hongyu Yu

**Affiliations:** 1 School of Integrated Circuits and Electronics, Beijing Institute of Technology, Beijing, China; 2 Department of Mechanical and Aerospace Engineering, The Hong Kong University of Science and Technology, Kowloon, Hong Kong China

**Keywords:** Electrical and electronic engineering, Carbon nanotubes and fullerenes

## Abstract

Wearable strain sensors have been widely used for human activity monitoring. Most reported strain sensors have mainly focused on material engineering, high stretchability and large gauge factors. Few works have focused on strain sensor’s robustness and reliability, including low hysteresis, good long-term stability, good electrode material stability, and low coupling effects under multi-input signals, which are the factors that limit practical strain sensor applications. To develop a high-performance strain sensor, we propose a flexible capacitive sensor structure with three-dimensional (3D) interdigital electrodes fabricated by vertically aligned carbon nanotubes. Compared with a traditional resistive strain sensor and a capacitive strain sensor with vertical sandwich electrodes, a strain sensor with horizontal parallel interdigital electrodes can benefit from low cross talk in terms of the normal force and improve substrate transparency. Additionally, embedding 3D electrodes into the substrate improves ultrahigh robustness with a low-pressure coupling effect under normal force. Moreover, compared with other reported works, the electrode variation under strain is small (less than 1.6%), which means that the perturbation of inert properties on device performance is small. Finally, the fabricated strain sensor achieves an ultralow hysteresis (0.35%), excellent pressure-insensitive performance (less than 0.8%), fast response (60 ms), good long-term stability, and good transparency. As an application example, a flexible strain sensor was successfully demonstrated as a wearable device for the precise monitoring of different types of human activities, including bending of the finger, knee, elbow, wrist, and neck with large strain signals and small strain signals generated by a mouth-opening activity. This excellent performance indicates that the flexible strain sensor is a promising candidate for human motion detection, soft robotics, and medical care.

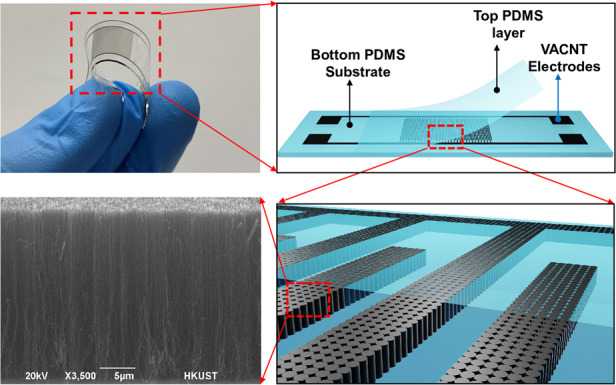

## Introduction

The development of wearable electronics has attracted tremendous interest in recent years with versatile applications in human motion detection, soft robotics, electronic skin, and human‒machine interfaces^1–5^. Stretchable and flexible strain sensors can transduce mechanical deformations into electric signals, which enables the detection of human motion activity^1,6–9^, such as joint bending and vocalization vibration. To meet the requirements of the above applications, strain sensors with low hysteresis, fast response, high stretchability, and good long-term reliability are needed^3,10–15^. Furthermore, strain sensors must be transparent and not obscure the transmission of light^16,17^, which can be useful when integrating photosensitive components (e.g., UV monitoring, photocell power supply, phototherapy) for medical diagnosis and avoiding decreases in melanin secretion caused by shading. More importantly, users of strain sensors on body surfaces, including faces and joints, prefer sensors with high transparency and low visibility during activities^18^.

The materials used for both stretchable substrates and electrodes are diverse. Polydimethylsiloxane (PDMS), with soft deformation and better light transmittance properties, is frequently used as a flexible substrate^16,19^. Ecoflex^20^ and hydrogel^21,22^ have excellent stretchability and are mostly applied to fabricate large stretchable strain sensors (*ε* > 100%). In addition, other stretchable materials, including stretchable elastomers (epoxy aliphatic acrylate and aliphatic urethane diacrylate)^23^, polyvinylidene fluoride (PVDF)^6^, thermoplastic polyurethane (TPU)^12^, natural rubber latex^24^, styrene-(ethylene-butylene)-styrene (SEBS)^25^, and polyimide^26^, are also utilized for stretchable substrates. For stretchable electrodes, carbon materials such as carbon black^19^, graphene nanoplates^10^, carbon nanotubes^23^, and graphene^24^ are preferable for work with elastomers due to their better conductivity, low cost, and easy fabrication process. In addition, silver nanoparticles^16^, liquid metal^20^, Au^26^, and Ag nanowire/MXene composites^27^ have also been utilized in specific designs for conductive electrodes. Based on different sensing mechanisms, strain sensors can be divided into several categories, including optical^28^, capacitive^10,16^, piezoelectric^29,30^, and electrical resistive strain sensors^23,31^. Optical strain sensors rely on the light transmittance change of the sensing material under different strain conditions, and additional light intensity detection components are needed. Piezoelectric strain signals result from the force between two electrodes under bending or stretching deformation, and the sensing material and electrodes can limit applications over a large measurement range^1,30^. Electrical resistive strain sensors are the most common type due to their simple structure and fabrication process^3,5^. However, the resistance change of the sensor not only results from the geometric structure changing but also depends on the conductivity of the material under applied changing strain, which can lead to unsatisfactory linearity and extreme hysteresis behaviors^16^. In resistive strain sensors, the strain signal has a significant coupling effect with the pressure signal, which makes it difficult to achieve accurate measurement results. In contrast, the capacitance change of a capacitive strain sensor mainly relies on the geometry change of the dielectric material and the sensing electrode, which can benefit a sensor with good linearity and low hysteresis. Currently, capacitive strain sensors are mainly designed with a vertical sandwich structure, which blocks light and reduces substrate transparency performance^32^. Moreover, the electrode materials are not stable because of cracks appearing during the stretching process, and can lead to inaccurate measurement results^16^.

Herein, a novel stretchable transparent capacitive strain sensor with interdigital three-dimensional (3D) electrodes fabricated by vertically aligned carbon nanotubes is proposed. The capacitive strain sensor consists of interdigital electrodes on a stretchable PDMS insulator substrate to achieve high sensitivity, transparency, linearity, and reduced hysteresis. The interdigital electrodes can reduce sensor thickness compared with a traditional capacitive strain sensor with vertical electrodes. Additionally, the proposed strain sensor also exhibits an excellent dynamic response, including fast response, long-term stability, and robustness. More importantly, the strain sensor is pressure-insensitive and can provide more accurate measurement results and reduce the cross talk with multiple excitation signals. These characteristics enable the use of the proposed strain sensor as a wearable device to monitor various human activities, including large-scale motions (finger bending, knee bending, neck bending, wrist bending, and elbow bending) and small-scale motions (mouth opening).

## Results

### Device fabrication

**Fig. 1:**
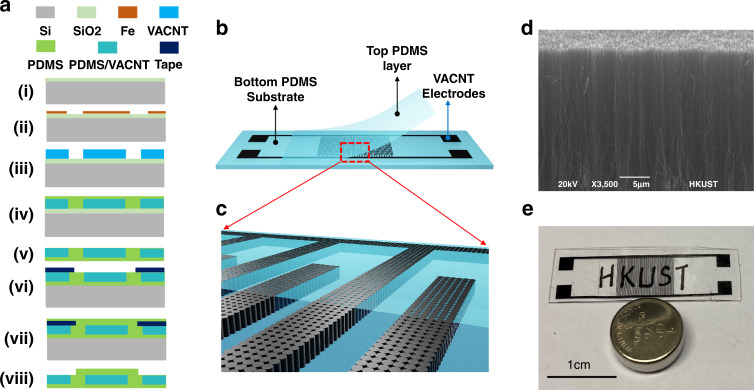
The process flow and structure schematics of the strain sensor. **a** Schematic of the fabrication process of a stretchable transparent strain sensor with 3D electrodes fabricated by vertically aligned carbon nanotubes. **b** Exploded view of the flexible strain sensor. **c** Enlarged view of the vertically aligned carbon nanotube electrodes. **d** SEM image of the vertically aligned carbon nanotubes. **e** Image of the fabricated strain sensor showing its transparent performance

**Fig. 2:**
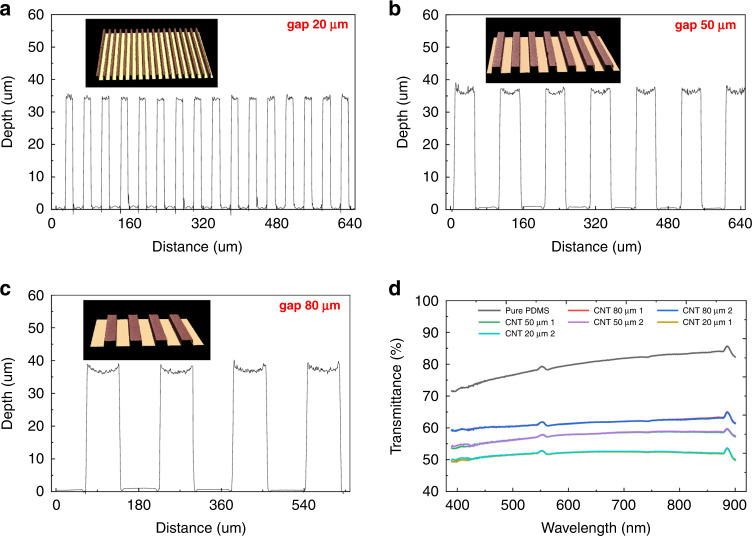
Demonstration of transmittance of strain sensor. The surface profile of the three-dimensional electrodes fabricated by the vertically aligned carbon nanotubes with three types of gaps and width dimensions: **a** 20 μm, **b** 50 μm, and **c** 80 μm. **d** Optical transparency results of pure PDMS and PDMS-CNT structures with interdigital electrode gaps of 20, 50, and 80 μm

**Fig. 3:**
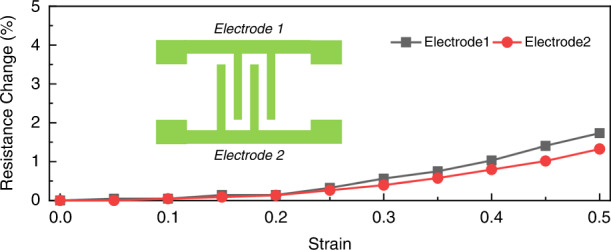
The insensitivity of electrode resistance to strain. Electrode resistance change of the strain sensor under the stretching process

A schematic of the fabrication flow process of the stretchable capacitance strain sensor is shown in Fig. 1a. A 2-nm Fe thin-seed layer was grown on a silicon wafer with a 1-µm silicon oxide isolation layer on top (Fig. 1a (i)) using an E-beam evaporator and patterned for an interdigital finger shape with a lift-off process (Fig. 1a (ii)). Then, a vertically aligned carbon nanotube (VACNT) forest (Fig. 1a (iii)) was synthesized using a microwave plasma-enhanced chemical vapor deposition (PECVD) apparatus to form the sensor electrodes. A degassed PDMS precursor mixer (monomer/curing agent: 10/1, purchased from Dowsil) was spun on the surface of the wafer to cover the VACNT using a spin coater with a rotation speed of 150 rotations/min for 40 s (Fig. 1a (iv)). To reduce gas bubbles in the PDMS layer and enhance the PDMS filling performance in the gaps of the carbon nanotubes, the wafer was placed into a vacuum chamber and vacuumed for 20 min. During this procedure, the vertically aligned carbon nanotubes were embedded into the PDMS to form 3D electrodes. Then, the PDMS was cured on a hot plate for 2 h at 70 °C and then used to peel off the VACNT electrodes from the silicon wafer. The thickness of the PDMS was characterized as 400 µm. After peeling off (Fig. 1a (v)), the PDMS substrate with 3D electrodes on one side was attached to the silicon wafer again on the other side (Fig. 1a (vi)). To protect the top side of the CNT electrodes, an additional PDMS layer was coated with a rotation speed of 2000 rotations/min for 40 s (Fig. 1a (vii)) and cured on a hot plate at 70 °C for 2 h. Before coating the PDMS, a piece of masking tape was cut and placed on top of the device to prevent the PDMS from coating the signal connection pads. Finally, the tape was removed to expose the pads, and the strain sensor was peeled off from the substrate (Fig. 1a (viii)). A schematic of an exploded view of the flexible strain sensor is shown in Fig. 1b, and an enlarged view of the vertically aligned carbon nanotube electrodes is shown in Fig. 1c. Figure 1d shows an SEM image of the vertically aligned carbon nanotubes. An image of the sensor is shown in Fig. 1e, demonstrating its size and transparent performance. The vertically aligned carbon nanotube-based electrodes were patterned with three gap and width dimensions—20, 50, and 80 μm—for comparison, where the width and gap were kept at the same value. The height of the 3D electrodes was characterized by a Keyence 3D laser confocal microscope, which is shown in Fig. 2. All the electrodes showed good uniformity and a superior vertical profile. The height of the 3D electrodes was ~35 μm. The transparency performance of pure PDMS and PDMS with interdigital CNT electrodes was tested from 380 to 900 nm. The transparency of the pure PDMS at 550 nm wavelength was ~79% (Fig. 2d). The transparency of devices with CNT electrodes at 550 nm was 61.6% (with an electrode gap of 80 μm), 57.6% (with an electrode gap of 50 μm), and 52.7% (with an electrode gap of 20 μm). The larger the gap size of the electrodes was, the more transparent the substrate, which resulted from less light being blocked by the CNT electrodes. The 3D electrode resistance of the strain sensor during the stretching process was highly stable (Fig. 3), at less than 1.6% variation for the full strain range (ε = 50%). A stable electrode resistance indicates that there were no cracks generated during stretching, ensuring long-term stability and robustness.

### Theoretical modeling

**Fig. 4:**
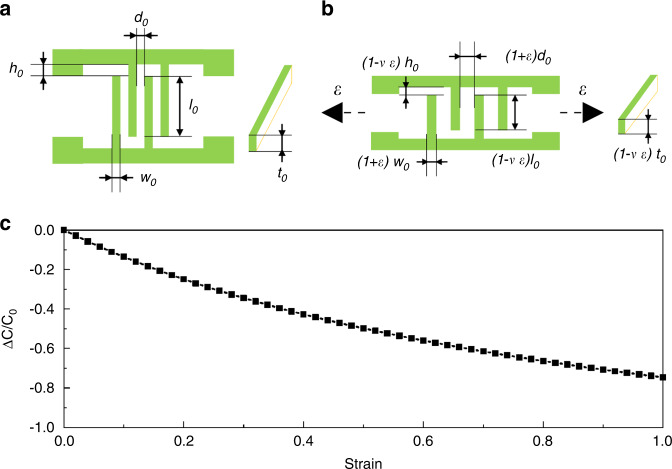
Geometric model of strain sensor and theoretical analysis of capacitance variation. Schematic of the interdigital electrode-based capacitance strain sensor-labeled critical dimension parameters under **a** initial condition and **b** stretching condition. **c** Normalized sensitivity ΔC/C_0_ of the capacitive strain sensor as a function of the strain (l_0_ = 50 μm, d_0_ = 100 μm, h_0_ = 100 μm, w_0_ = 50 μm, v = 0.5)

Figure 4a shows the key parameters of the strain sensor without strain. The capacitance of the total multiple electrodes can be calculated by the following equations:

1 $$\left\{ \begin{array}{l}\!\!{{{\mathrm{C}}}}_0 = {{{\mathrm{C}}}}^\prime + C{^{\prime\prime}} \\ \!\!C^\prime = \lambda \frac{{l_0t_0}}{{d_0}}(n - 1)\\\!\! C{^{\prime\prime}} = \lambda \frac{{w_0t_0}}{{h_0}}(n - 1)\end{array} \right.$$

where λ represents the absolute permittivity of the polydimethylsiloxane; *l*_*0*_, *t*_*0*_, *h*_*0*_, *d*_*0*_, and *w*_0_ are the length, height, gap between parallel electrode terminals and backbone electrodes, the gap between parallel electrodes, and width of the 3D electrodes, respectively; *n* is the number of total electrodes of the device, and C′ and C″ are the capacitance between the parallel electrodes and the capacitance between the end of the parallel electrode and the backbone electrode. For capacitance C″, the total number of subcapacitors is *n*, which is recognized as *n*-1 for simplification because *w*_*0*_ is two orders of magnitude smaller than *l*_*0*_. The capacitance change of the device under strain in the perpendicular direction of the electrodes is shown in Fig. 4b. Based on the properties of the elastic material, dimensions including *d*_*0*_ and *w*_0_ in the *x*-direction can stretch to (1 + *ε*) *d*_*0*_ and (1 + *ε*) *w*_0_, dimensions including *l*_*0*_ and *h*_*0*_ in the y-direction can shrink to (1−*εv*) *l*_*0*_, and (1−*εv*) *h*_*0*_ and dimensions including *t*_*0*_ in the z-direction can shrink to (1−*εv*) *t*_*0*_. The capacitance of the stretched sensor can be calculated with Eq. (2):

2 $${{{\mathrm{C}}}} = \lambda (n - 1)\left(\frac{{(1 - \nu \varepsilon )l_0(1 - \nu \varepsilon )t_0}}{{(1 + \varepsilon )d_0}} + \frac{{(1 + \varepsilon )w_0(1 - \nu \varepsilon )t_0}}{{(1 - \nu \varepsilon )h_0}}\right)$$

where *v* is the Poisson’s contraction ratio of PDMS and *ε* is the strain of the sensor under stretching. The sensitivity of the capacitive strain sensor can be expressed by the following equation:

3 $$\frac{{\Delta C}}{{C_0}} = \frac{{C - C_0}}{{C_0}} = \frac{{\left( {\frac{{(1 - \nu \varepsilon )^2l_0}}{{(1 + \varepsilon )d_0}} + \frac{{(1 + \varepsilon )w_0}}{{h_0}}} \right)}}{{\left( {\frac{{l_0}}{{d_0}} + \frac{{w_0}}{{h_0}}} \right)}} - 1$$

Based on Eq. (3), the normalized sensitivity of the capacitive strain sensor can be plotted in Fig. 4c with assumed dimensions of *l*_*0*_ = 50 μm, *d*_*0*_ = 100 μm, *h*_*0*_ = 100 μm, and *w*_*0*_ = 20 μm. The soft substrate PDMS is regarded as an elastic material with a Poisson’s ratio of *v* = 0.5. The strain range is analyzed from 0 to 1.

### Performance characterization

**Fig. 5:**
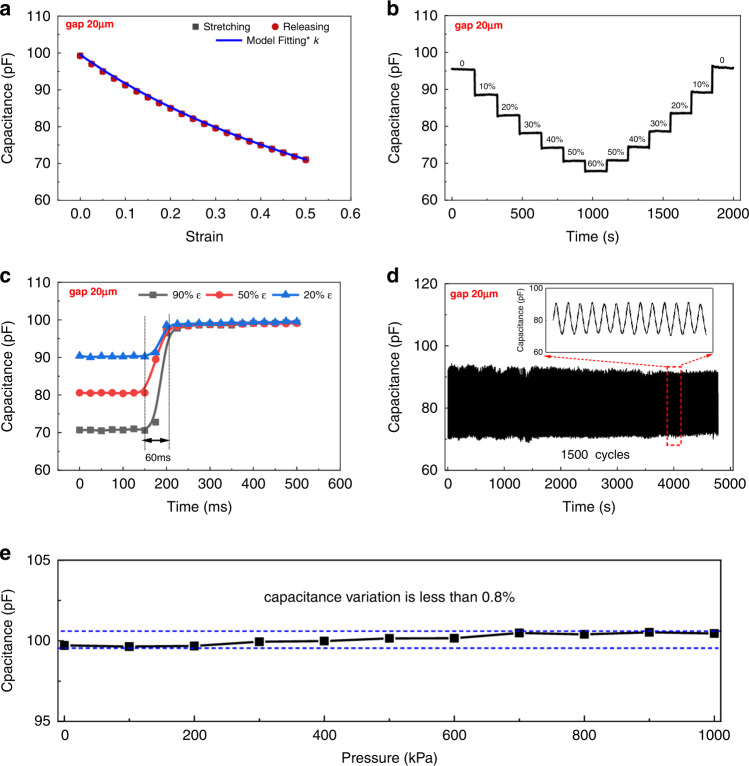
Performance calibration of strain sensor. **a** Output and hysteresis results of the strain sensor fitted with the theoretical model (*k* = 0.57). **b** Test results of the sensor with step input signals for both stretching and releasing processes. **c** Transient test results of the sensor with three types of strain variations, including 90% ε, 50% ε, and 20% ε. **d** Long-term stability test results of the strain sensor from 20% ε to 90% ε (inset is the enlarged figure for a certain period). **e** Pressure effect on the sensing performance of the strain sensor. The capacitance variation is less than 0.8% for the whole measurement range (0–1 MPa)

The fabricated strain sensors were first characterized to check the primary performance with the stretching test setup (Fig. S1). First, the sensitivity and hysteresis were tested with a strain sensor with a gap of 20 μm, as shown in Fig. 5a. The results of two other strain sensors with gaps of 50 and 80 μm are shown in Fig. S2a, b. The gauge factors (GFs) of the strain sensors were 0.637 (20 μm), 0.505 (50 μm), and 0.413 (80 μm), which can be calculated by the following equation:

4 $${\mathrm{GF}} = \frac{{\Delta C/C_0}}{\varepsilon }$$

From the results, we can see that the smaller the gap size is, the higher the gauge factor. Combined with the transparency performance of the device shown in Fig. 2d, the gap size of the electrodes needs to be designed for different applications to balance the gauge factor and transparency. Hysteresis represents the consistent performance during the stretching and releasing process for the same strain value. Low hysteresis can provide a more accurate measurement regardless of the stretch or release process. The hysteresis is characterized as seen in Fig. 5a, which shows that the device has an ultralow hysteresis value for the whole measurement range. The maximum hysteresis of the three strain sensors was 0.35% (20 μm, Fig. 5a), 0.26% (50 μm, Fig. S2b), and 0.13% (80 μm, Fig. S2c). We also checked the validity range of the proposed model, and the result shows that the model can provide convincing consistency in a normalized strain range of 0–100% (Fig. S2a). In addition, the sensor was fitted with the proposed theoretical model, which indicates good agreement with the test results with a fitting factor k (C_test_/C_model_ = 0.57). The purpose of using semiempirical factors is to eliminate the errors caused by the complex fringe electric field, parasitic capacitance, material property, and electrode deformation. Figure 5b shows the test results with a step input strain signal both for the stretching and releasing processes, which represents good short-term stability. Two other sensors were also characterized, as shown in Fig. S3a (50 μm) and Fig. S3b (80 μm). The transient response of the strain sensor was characterized by three strain variations, including 90% ε, 50% ε, and 20% ε. The sensor was first stretched at a certain strain value, followed by a sudden release of the terminal. The response time of the strain sensor was calculated from the release point to 90% of the whole strain variation. Figure 5c shows that the response time of the sensor was ~60 ms, which is good enough for most applications in human activity monitoring. Strain sensors with electrode gaps of 50 and 80 μm were also characterized with a similar response time of 60 ms (Fig. S4a, b) because all the substrates were the same material with the same thickness. Long-term stability was measured with 1500 testing cycles with no significant performance attenuation (Fig. 5d). The fabricated strain sensor was also characterized by the pressure effect on the capacitance variation (<0.8%) of the strain sensor, which is shown in Fig. 5e. The results show that our sensor has an excellent pressure-insensitive performance with pressures ranging from 0 Pa to 1 MPa, which can be of benefit for more applications and provide more accurate data.

### Human activity monitoring

**Fig. 6:**
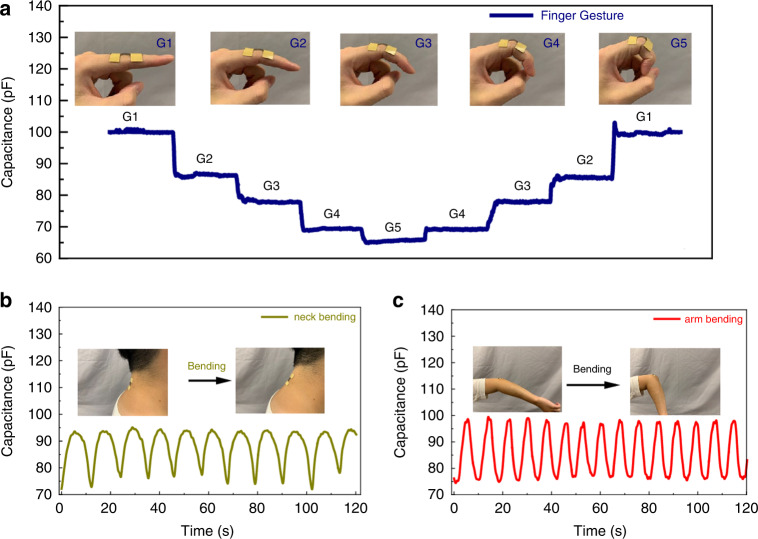
Detection of large strain signal by the sensor. Human joint gesture test results include **a** finger, **b** neck, and **c** elbow bending

The human‒machine interface is a significant application area that requires many sensors to transfer human motion and gestures to electric signals for machine control, such as in robotics. Our proposed high-performance strain sensor shows the possibility for use in various potential applications, including large-scale human motion detection, given its stable large dynamic response to neck bending, finger gestures, elbow bending, knee bending, and wrist bending, which is shown in Fig. 6. The finger test is shown in Fig. 6a. Five gestures can be monitored for different bending positions of the finger joints. Slow bending and fast bending measurements are demonstrated by the neck joint (Fig. 6b) and wrist joint (Fig. 6c), respectively. Additionally, the leg bending and wrist bending results are also recorded, which are shown in Fig. S5a,b.

**Fig. 7:**
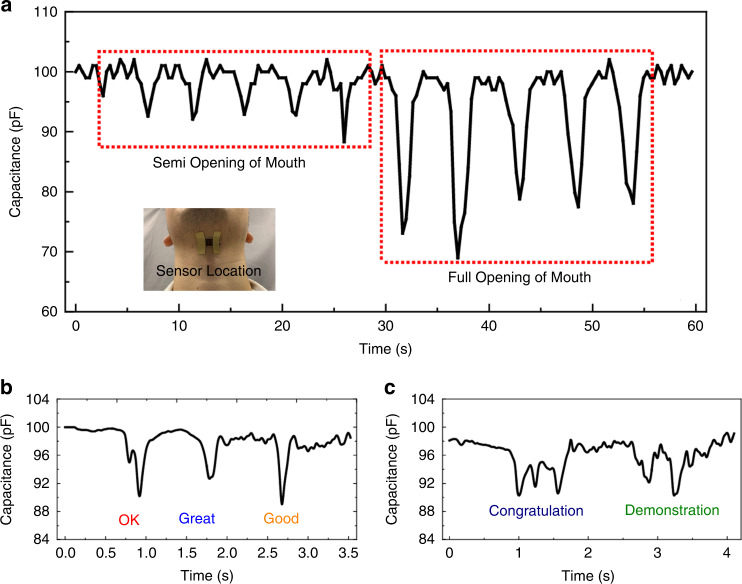
Detection of small strain signal by the sensor. **a** Characterization of the sensing performance of the strain sensor used for mouth open monitoring. **b** Voice testing based on open mouth detection for simple words. **c** Voice monitoring based on open mouth recognition for complex words

For some special activities, such as a smile, eye-opening, and mouth opening, the displacement of these areas is quite small. A stable strain sensor with high sensitivity is required for movement monitoring of these typical applications. Normal speaking and mouth movements can cause skin variations under the throat. A strain sensor mounted on the throat can monitor these tiny movements. Figure 7a shows the mouth opening test results, including semi-opening of the mouth and full opening of the mouth, which illustrates that an increased mouth opening can provide a larger strain signal (in other words, larger capacitance change). Simple words are tested with this high-performance strain sensor, including “OK”, “Great”, and “Good” (Fig. 7b). For these simple words, the mouth undergoes only one large opening process. For some complex words, including “Congratulations” and “Demonstration”, the mouth-opening process contains multiple peaks (Fig. 7c). Thus, this sensing behavior can be used on special occasions. For example, it can be used for mouth-opening detection of a patient with speaking problems and to send an alarm signal to doctors. It can also help people with deafness correct their pronunciation by tuning the mouth-opening size.

## Conclusion

We successfully fabricated a stretchable strain sensor with 3D vertically aligned carbon nanotube-based interdigital electrodes. Compared with a strain sensor using a conventional vertical sandwich capacitor, our device uses an in-plane parallel electrode that can reduce the substrate thickness, improve the cross talk performance resulting from the normal force, and gain good transparency with the transparent material PDMS. The electrode experiences a small resistance variation (<1.6%) for the whole stretch range (ε = 0.5), which enables stable connection performance and reduces its interference on the readout circuit of the sensor. The sensor also demonstrates an excellent pressure-insensitive performance of up to 1 MPa load that improves reliability and reduces the cross talk from the pressure signal during the stretching and releasing process. The sensor provides an ultralow hysteresis of less than 0.35%, which can provide accurate and reliable stretching and release measurement data. The device was tested for 1500 cycles under a measurement range from 20% ε to 90% ε and showed stable performance. With excellent performance, the device has demonstrated usefulness in many potential applications, such as human activity monitoring, both for large strain signals generated by joint bending (knee, elbow, leg, finger, and neck) and for small strain signals created by mouth opening. All of these demonstrations make it a promising wearable device that can be used for human‒machine interfaces, health care, and soft robotics.

## Methods

### Fabrication of the vertically aligned carbon nanotubes

A microwave plasma-enhanced chemical vapor deposition (PECVD) apparatus was used for CNT growth with the following details. After the PECVD reaction chamber was evacuated, 50 sccm nitrogen (N_2_) and 10 sccm hydrogen (H_2_) were introduced into the chamber. Then, the sample in the chamber was heated to 800 °C. After stabilization, methane (CH_4_) was injected into the chamber at a flow rate of 30 sccm for 60 s, and microwave plasma power was lit up with a power of 200 W at a gas pressure of 8 Torr. During this process, CNTs precipitated from the catalyst particles following the top growth theory. After growth, the chamber was cooled to room temperature with 50 sccm N_2_ and 40 sccm H_2_. The length of the vertical CNTs was ~35 μm, which can be controlled by adjusting the growing time.

### General characterization techniques

A Gwinstek LCR meter 6020 (RS company) was used for the data recording, including the electrode resistance change under the stretching process, capacitance change under pressure, performance characterization (sensitivity, hysteresis, short-term reliability, long-term reliability, response time), and human activity demonstration (finger bending, elbow bending, neck bending, leg bending, knee bending, and mouth opening). The three-axis displacement platform SELN LY40-L (Dongguan Shengling Precision Machinery Co. Ltd.) was used to test the sensitivity, hysteresis performance, and electrode resistance of the sensor. A cycling test of the strain sensor was characterized by the stretching platform C-beam (Openbuilds). Pressure effect testing was driven by an HLD electric test stand machine (Handpi). A UV/VUS spectrophotometer (Perkin Elmer) was used to characterize the transparency performance of the strain sensor from 380 to 900 nm wavelengths.

## Supplementary information

Supplementary Information

## Data Availability

The experimental data referenced in this text are available from the authors upon reasonable request.
