# Supplementary material for: Low-hysteresis, pressure-insensitive, and transparent capacitive strain sensor for human activity monitoring
Source: Microsyst Nanoeng. 2022 Oct 12;8:113. doi: 10.1038/s41378-022-00450-7 (PMC9553868; doi:10.1038/s41378-022-00450-7)
Supplement: Supplementary file 1 — Supplementary Information [file 41378_2022_450_MOESM1_ESM.docx]

**Supplementary information**

# Low-hysteresis, Pressure-insensitive, and Transparent Capacitive Strain Sensor for Human Activity Monitoring

*Xiaoyi Wang^1 ,2,^*, Yang Deng^2^, Peng Jiang^2^, Xingru Chen^2^, Hongyu Yu^2,^**

^1^School of Integrated Circuits and Electronics, Beijing Institute of Technology, Beijing, China

^2^Department of Mechanical and Aerospace Engineering, Hong Kong University of Science and Technology, Kowloon, Hong Kong, China

**Corresponding authors:**

Prof. Hongyu Yu

Email: [hongyuyu@ust.hk](mailto:hongyuyu@ust.hk)

Telephone: +852 34692754

Prof. Xiaoyi Wang

Email: xiaoyiwang@bit.edu.cn

Telephone: +86 15868802615

**Fig. S1.** Testing setup of the strain sensor for gauge factor and hysteresis characterization.

**Fig. S2.** (**a**) The normalized strain range of 0–100% with a gap of 20 μm. Test results of the strain sensor with gaps of (**b**) 50 μm and (**c**) 80 μm under the stretching and releasing process.

**Fig. S3.** Stable test results of the sensors with gaps of (**a**) 50 μm and (**b**) 80 μm with a step input signal for both the stretching and releasing processes.

**Fig. S4.** Transient results of the sensor with three types of strain variations, including 90% ε, 50% ε, and 20% ε, for gaps of (a) 50 μm and (b) 80 μm.

**Fig. S5**. Test results of (**a**) leg bending and (**b**) wrist bending.
